# Lipoproteins act as vehicles for lipid antigen delivery and activation of invariant natural killer T cells

**DOI:** 10.1172/jci.insight.158089

**Published:** 2023-05-08

**Authors:** Suzanne E. Engelen, Francesca A. Ververs, Angela Markovska, B. Christoffer Lagerholm, Jordan M. Kraaijenhof, Laura I.E. Yousif, Yasemin-Xiomara Zurke, Can M.C. Gulersonmez, Sander Kooijman, Michael Goddard, Robert J. van Eijkeren, Peter J. Jervis, Gurdyal S. Besra, Saskia Haitjema, Folkert W. Asselbergs, Eric Kalkhoven, Hidde L. Ploegh, Marianne Boes, Vincenzo Cerundolo, G.K. Hovingh, Mariolina Salio, Edwin C.A. Stigter, Patrick C.N. Rensen, Claudia Monaco, Henk S. Schipper

**Affiliations:** 1 Kennedy Institute of Rheumatology, University of Oxford, Oxford, United Kingdom.; 2 Center for Translational Immunology, University Medical Center Utrecht, Utrecht, Netherlands.; 3 Wolfson Imaging Centre, Medical Research Council (MRC) Weatherall Institute of Molecular Medicine, University of Oxford, Oxford, United Kingdom.; 4 Department of Vascular Medicine, Amsterdam Medical Center, Amsterdam, Netherlands.; 5 Center for Molecular Medicine, University Medical Center Utrecht, Utrecht, Netherlands.; 6 Division of Endocrinology and Einthoven Laboratory for Vascular and Regenerative Medicine, Department of Medicine, Leiden University Medical Center, Leiden, Netherlands.; 7 Institute of Microbiology and Infection, School of Biosciences, University of Birmingham, Birmingham, United Kingdom.; 8 Central Diagnostic Laboratory and; 9 Division of Heart & Lungs, Department of Cardiology, University Medical Center Utrecht, Utrecht, Netherlands.; 10 Institute of Cardiovascular Science and Institute of Health Informatics, Faculty of Population Health Sciences, University College London (UCL), London, United Kingdom.; 11 Boston Children’s Hospital and Harvard Medical School, Boston, Massachusetts, USA.; 12 MRC Weatherall Institute of Molecular Medicine, University of Oxford, Oxford, United Kingdom.; 13 Department of Pediatric Cardiology, Wilhelmina Children’s Hospital, University Medical Center Utrecht, Utrecht, Netherlands.

**Keywords:** Immunology, Lipoproteins, NKT cells

## Abstract

Invariant natural killer T (iNKT) cells act at the interface between lipid metabolism and immunity because of their restriction to lipid antigens presented on CD1d by antigen-presenting cells (APCs). How foreign lipid antigens are delivered to APCs remains elusive. Since lipoproteins routinely bind glycosylceramides structurally similar to lipid antigens, we hypothesized that circulating lipoproteins form complexes with foreign lipid antigens. In this study, we used 2-color fluorescence correlation spectroscopy to show, for the first time to our knowledge, stable complex formation of lipid antigens α-galactosylceramide (αGalCer), isoglobotrihexosylceramide, and OCH, a sphingosine-truncated analog of αGalCer, with VLDL and/or LDL in vitro and in vivo. We demonstrate LDL receptor–mediated (LDLR-mediated) uptake of lipoprotein-αGalCer complexes by APCs, leading to potent complex-mediated activation of iNKT cells in vitro and in vivo. Finally, LDLR-mutant PBMCs of patients with familial hypercholesterolemia showed impaired activation and proliferation of iNKT cells upon stimulation, underscoring the relevance of lipoproteins as a lipid antigen delivery system in humans. Taken together, circulating lipoproteins form complexes with lipid antigens to facilitate their transport and uptake by APCs, leading to enhanced iNKT cell activation. This study thereby reveals a potentially novel mechanism of lipid antigen delivery to APCs and provides further insight into the immunological capacities of circulating lipoproteins.

## Graphical abstract

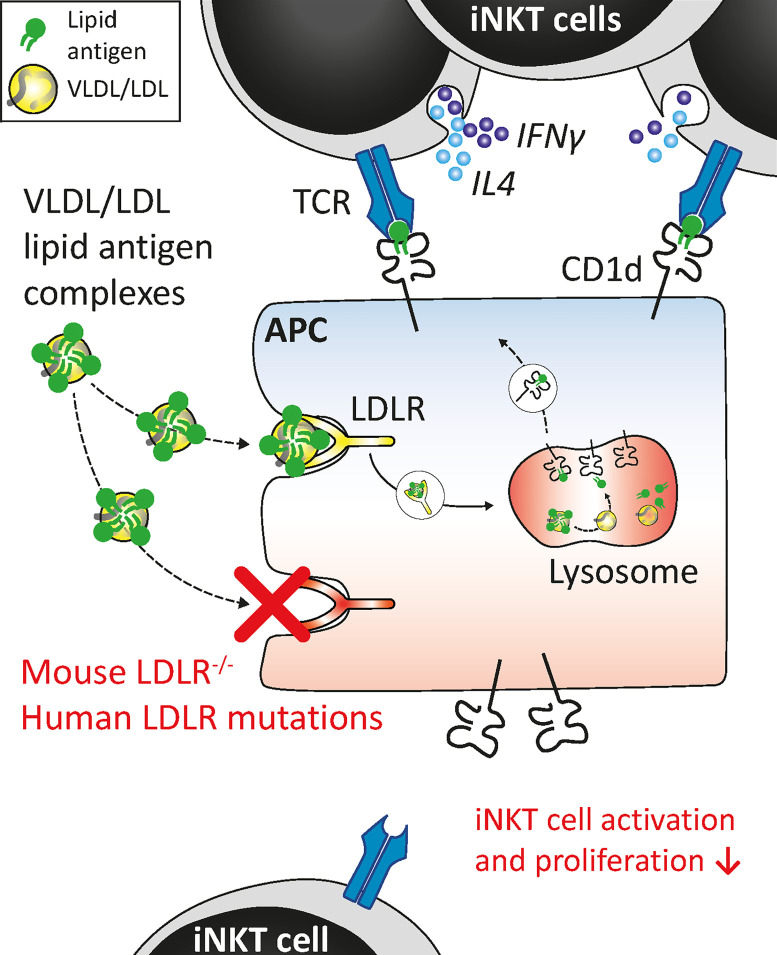

## Introduction

Invariant natural killer T (iNKT) cells are a specialized subset of T cells that recognize foreign and self lipid antigens presented by the MHCI-like molecule CD1d (1). Most of the foreign lipid antigens identified so far are glycolipids with a ceramide or glycerol backbone, such as *Sphingomonas*-derived glycosylceramides. These compounds structurally resemble the prototypic iNKT cell antigen α-galactosylceramide (αGalCer) (2). How foreign lipid antigens are delivered to antigen-presenting cells (APCs) remains elusive. Apolipoprotein E (apoE) is known to facilitate the uptake of lipid antigens by APCs (3, 4). Whether apoE is also involved in the transport and delivery of lipid antigens to APCs is currently unknown. In a recent patient study, we observed enhanced IFN-γ production by iNKT cells ex vivo upon supplementation of LDL (5). In contrast to VLDL and HDL, LDL does not bind apoE (6). Our data thus suggested an apoE-independent role for LDL in iNKT cell activation. The apoE-independent effects of LDL may be explained by biochemical studies, which have shown that glycosylceramides structurally similar to lipid antigens routinely bind to circulating lipoproteins (7, 8). In fact, glycosylceramides can insert into phospholipid rafts with a similar composition as the phospholipid shell of lipoproteins (9, 10). Therefore, we hypothesized that LDL and other lipoproteins can form complexes with lipid antigens and thus fulfill a distinct role in lipid antigen transport and uptake.

In this study, we investigated the role of lipoproteins in lipid antigen transport and delivery to APCs in vitro and in vivo. We used 2-color fluorescence correlation spectroscopy (2c-FCCS) to demonstrate complex formation of the prototypic lipid antigen αGalCer with VLDL and LDL in vitro and in vivo. Additionally, complex formation of LDL was demonstrated with the alternative lipid antigens isoglobotrihexosylceramide (iGb3) and OCH, a sphingosine-truncated analog of αGalCer. Upon LDL receptor–mediated (LDLR-mediated) uptake, lipid antigen-lipoprotein complexes potently activated iNKT cells in vitro. Administration in vivo of αGalCer-lipoprotein complexes likewise resulted in LDLR-mediated uptake and activation of iNKT cells in mice. Finally, LDLR-mutant patient PBMCs showed impaired activation and proliferation of iNKT cells, underscoring the relevance of lipoproteins for lipid antigen delivery and iNKT cell activation in humans. These results reveal a potentially novel mechanism for lipid antigen delivery to APCs and provide further insight into the immunological role of lipoproteins.

## Results

### Lipoproteins form stable complexes with the prototypic iNKT cell ligand αGalCer.

**Figure 1:**
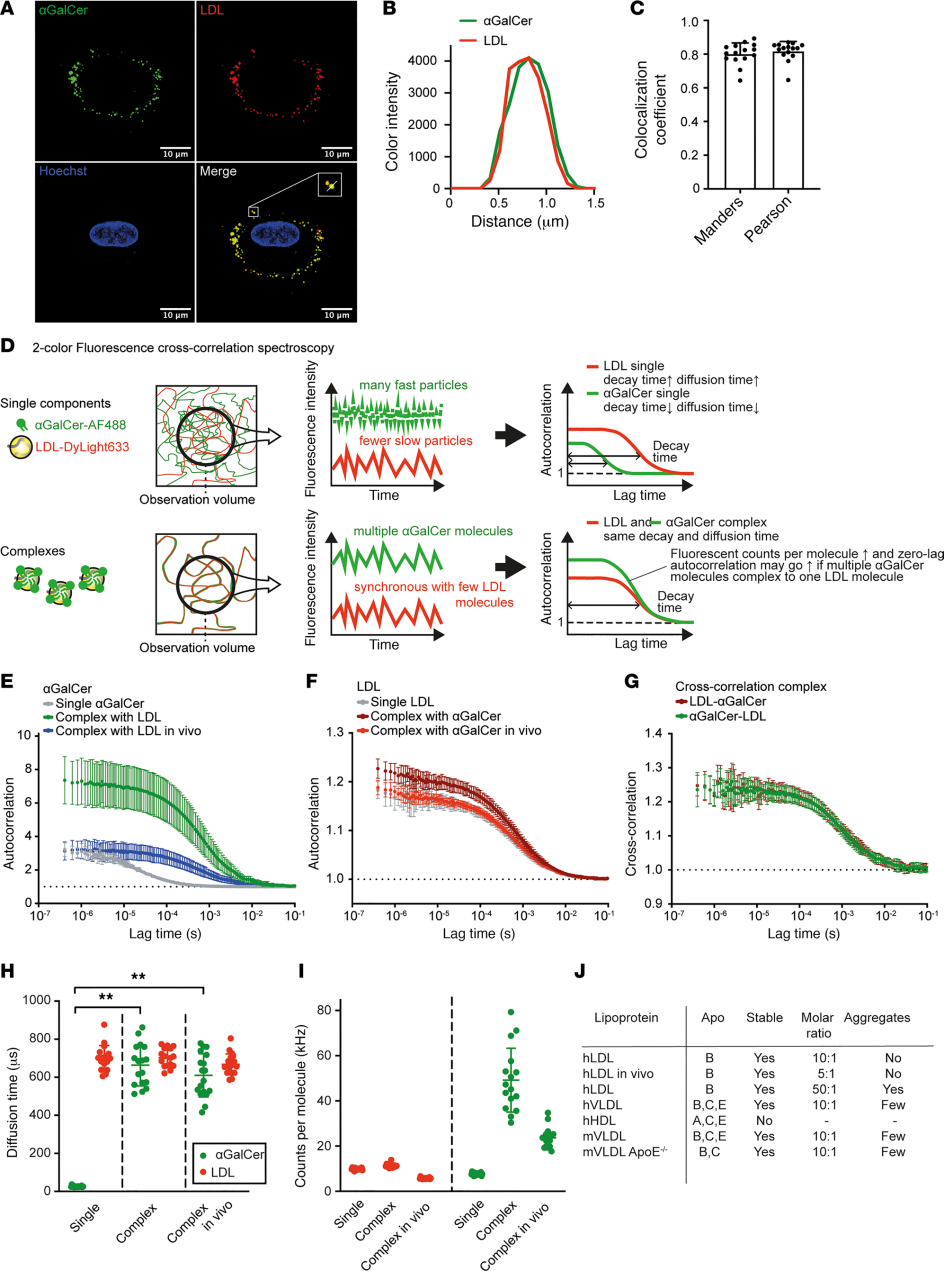
Lipoproteins form stable complexes with the prototypic iNKT cell ligand αGalCer. (**A**) Representative confocal image of αGalCer-AF488-LDL-DyLight 633 complexes loaded on human monocyte-derived macrophages. Scale bar: 10 μm. (**B**) Spatial colocalization of the αGalCer and LDL complex components (zoomed box in **A**). (**C**) Manders and Pearson colocalization coefficients of αGalCer and LDL (*n* = 15). (**D**) Principle of 2-color fluorescence cross-correlation spectroscopy (2c-FCCS). Diffusing particles are observed via their fluorescence. The measured fluorescence fluctuations over time are fitted in an autocorrelation model, which yields a decay time that reflects the average retention time of particles in the observation volume, as well as particle diffusion times. In case of complex formation, decay and diffusion times of the complex components will synchronize, and cross-correlated diffusion of the complex components will occur. (**E**) Autocorrelation curves of αGalCer singly and in complex with human LDL in vitro and in vivo, showing synchronization of αGalCer diffusion with LDL upon complexation (*n* = 15). (**F**) Autocorrelation curve of LDL in complex with αGalCer in vitro and in vivo (*n* = 15). (**G**) Cross-correlated diffusion of the αGalCer and LDL complex components (*n* = 15). (**H**) Diffusion time of single αGalCer and LDL, compared with complex components in vitro and in vivo (*n* = 15). (**I**) Fluorescent counts per molecule of single LDL and αGalCer and complexed components in vitro and in vivo. The αGalCer counts per molecule in vivo were normalized for the lower LDL counts per molecule in vivo (*n* = 15). (**J**) Overview of 2c-FCCS studies of lipoprotein-αGalCer complex formation. Statistics: error bars represent mean ± SD (**E**–**H**). Unpaired *t* tests with Bonferroni’s correction for multiple comparisons (**H**). **P < 0.01. Apo, apolipoprotein.

To investigate complex formation of lipoproteins with the prototypic iNKT cell ligand αGalCer, we coincubated fluorescently labeled human LDL and αGalCer for 1 hour, then loaded the LDL-αGalCer mixture onto human monocyte-derived macrophages. LDL is the dominant lipoprotein in humans and does not carry apoE (11–13), which allows us to study whether the lipoprotein-αGalCer interaction is apoE independent. Human LDL and αGalCer showed high spatial colocalization upon macrophage loading, in support of our hypothesis that lipoproteins form complexes with αGalCer (Figure 1, A–C). Therefore, we studied lipoprotein-αGalCer complex formation in more detail, using 2c-FCCS as an efficient means to assess stability and stoichiometry of the complexes (14, 15). In case of complex formation, autocorrelation curves of the diffusing complex components synchronize, and diffusion of the complex components shows cross-correlation (Figure 1D). After 1 hour of coincubation of fluorescently labeled αGalCer and human LDL in vitro at 37°C, autocorrelation curves of the diffusing complex components indeed synchronized (Figure 1, E and F). Cross-correlation studies verified complex formation (Figure 1G). The synchronization of αGalCer and LDL diffusion upon complex formation in vitro was also illustrated by the calculated diffusion times (Figure 1H). To assess complexation in vivo, fluorescently labeled human LDL and αGalCer were mixed in a syringe and instantly injected in an LDLR-deficient (*Ldlr^–/–^*) mouse, resulting in stable complex formation 30 minutes later (Figure 1, E, F, and H). Interestingly, analysis of the fluorescent αGalCer counts per molecule of LDL showed that in vitro complexation yielded binding of all available αGalCer molecules to human LDL in a 10:1 molar ratio, whereas in vivo complexation resulted in a molar ratio of 5:1 (Figure 1, I and J). Considering that all αGalCer molecules in vivo showed diffusion times similar to lipoproteins (Figure 1H), we assume that any remaining αGalCer has complexed with circulating mouse lipoproteins, which are structurally similar to human lipoproteins (12). To investigate whether other lipoprotein classes also form complexes with αGalCer, complex formation with human VLDL, human HDL, and mouse VLDL was assessed as well (Supplemental Figure 1, A–E; supplemental material available online with this article; https://doi.org/10.1172/jci.insight.158089DS1). HDL was the only lipoprotein that did not form complexes with αGalCer (Supplemental Figure 1C and Figure 1J). For subsequent in vitro and in vivo experiments, we therefore used LDL/VLDL-αGalCer complexes. To corroborate that lipoprotein-αGalCer complex formation is apoE independent, apoE-deficient mouse VLDL was subjected to complexation in vitro and showed similar complex formation with αGalCer as normal mouse VLDL (Supplemental Figure 1B and Figure 1J). Finally, complex stability was assessed by repeating 2c-FCCS after 1 week of storage at 4°C. Cross-correlation curves at day 0 and day 7 were compared and showed persistent cross-correlation of labeled αGalCer-488, and labeled human LDL-633, indicating stable complex formation (Supplemental Figure 2A). Taken together, LDL and VLDL form stable complexes with the prototypic iNKT cell ligand αGalCer, in an apoE-independent manner.

### LDL forms stable complexes with iGb3 and OCH.

**Figure 2:**
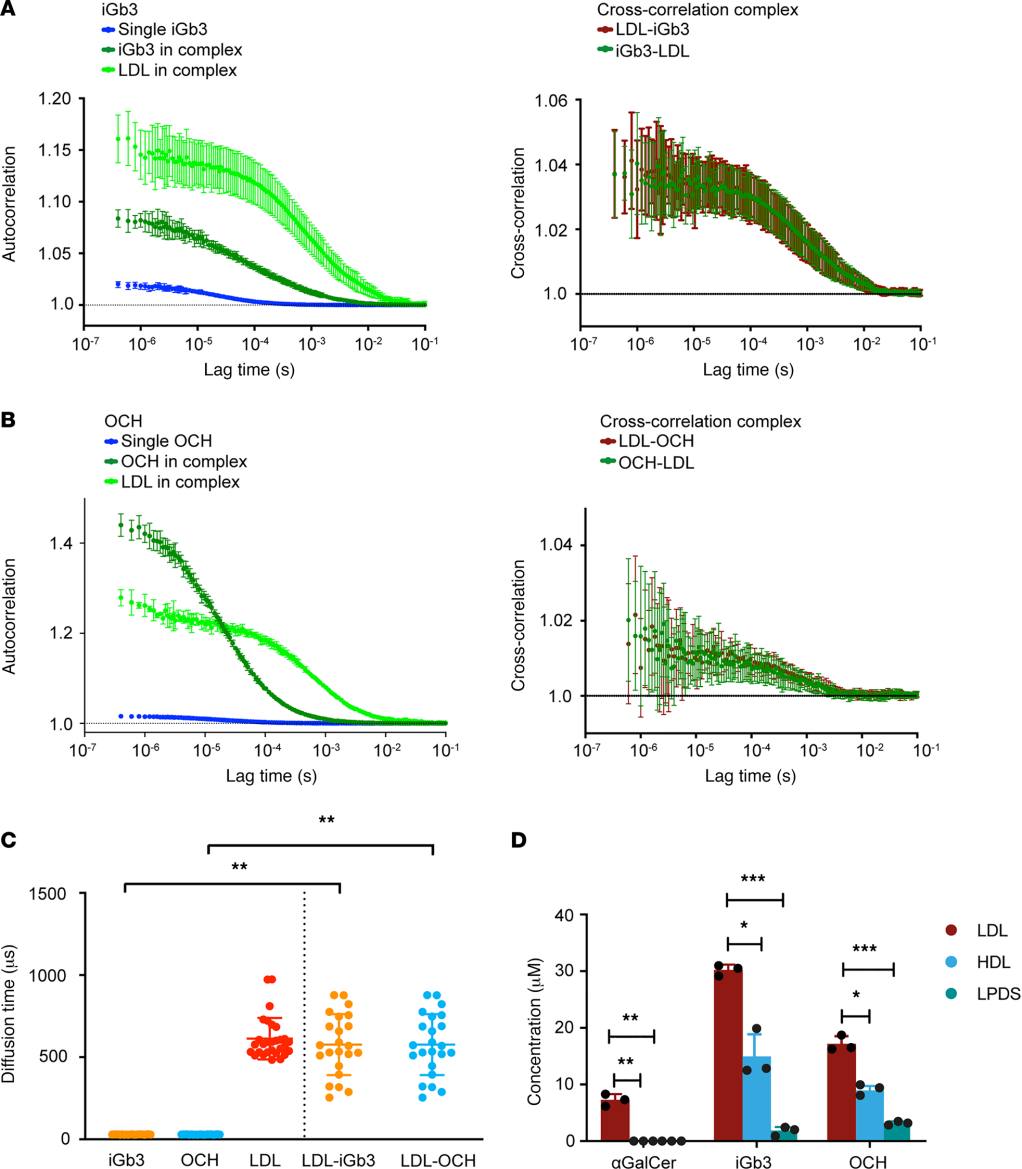
Lipoproteins form stable complexes with lipid antigens iGb3 and OCH. (**A**) Autocorrelation curves of DyLight 488–iGb3 singly and in complex with human DyLight 633–LDL in vitro, showing synchronization of iGb3 diffusion with LDL upon complexation (left panel). Cross-correlated diffusion of the iGb3 and LDL complex components (right panel) (*n* = 15). (**B**) Autocorrelation curves of DyLight 488–OCH singly and in complex with human DyLight 633–LDL in vitro, showing synchronization of OCH diffusion with LDL upon complexation (left panel). Cross-correlated diffusion of the OCH and LDL complex components (right panel) (*n* = 15). (**C**) Diffusion time of single iGb3, OCH, and LDL, compared with complex components in vitro (*n* = 15). (**D**) Concentration of lipid antigens αGalCer, iGb3, and OCH in different lipoprotein fractions isolated from human serum: LDL, HDL, and lipoprotein-depleted serum fraction (LPDS), measured by LC-MS (*n* = 3). Statistics: error bars represent mean ± SD. Unpaired *t* tests with Bonferroni’s correction for multiple comparisons (**C** and **D**). *P < 0.05; **P < 0.01; ***P < 0.001.

To assess whether complex formation with lipoproteins occurs with a wider variety of lipid antigens, complex formation of human LDL with 2 alternative lipid antigens that are widely used in literature, β-linked antigen iGb3 and OCH, a sphingosine-truncated analog of αGalCer, was assessed in a similar experimental setup (16). iGb3 and OCH were labeled using a DyLight 488 NHS Ester, then coincubated with human LDL at 37°C for 1 hour. 2c-FCCS showed that autocorrelation curves of the diffusing complex components synchronized, and cross-correlation curves verified complex formation for both iGb3 and OCH (Figure 2, A–C). Complex stability was assessed for iGb3 after 7 days and showed stable complex formation (Supplemental Figure 2B). OCH formed aggregates after 7 days and therefore stability was not assessed. To corroborate our findings in a human context, human serum was spiked with αGalCer, iGb3, and OCH, and lipoprotein fractions were isolated. Lipid antigen concentrations in the different fractions were measured using liquid chromatography-mass spectrometry (LC-MS) and indicated that the majority of lipid antigens were present in the LDL fraction (Figure 2D and Supplemental Figure 3, A and B). Together, LDL-cholesterol forms complexes with β-linked antigen iGb3 and αGalCer analog OCH, indicating that lipoproteins form complexes with a broad variety of lipid antigens.

### Uptake of lipoprotein-lipid antigen complexes and activation of iNKT cells in vitro.

**Figure 3:**
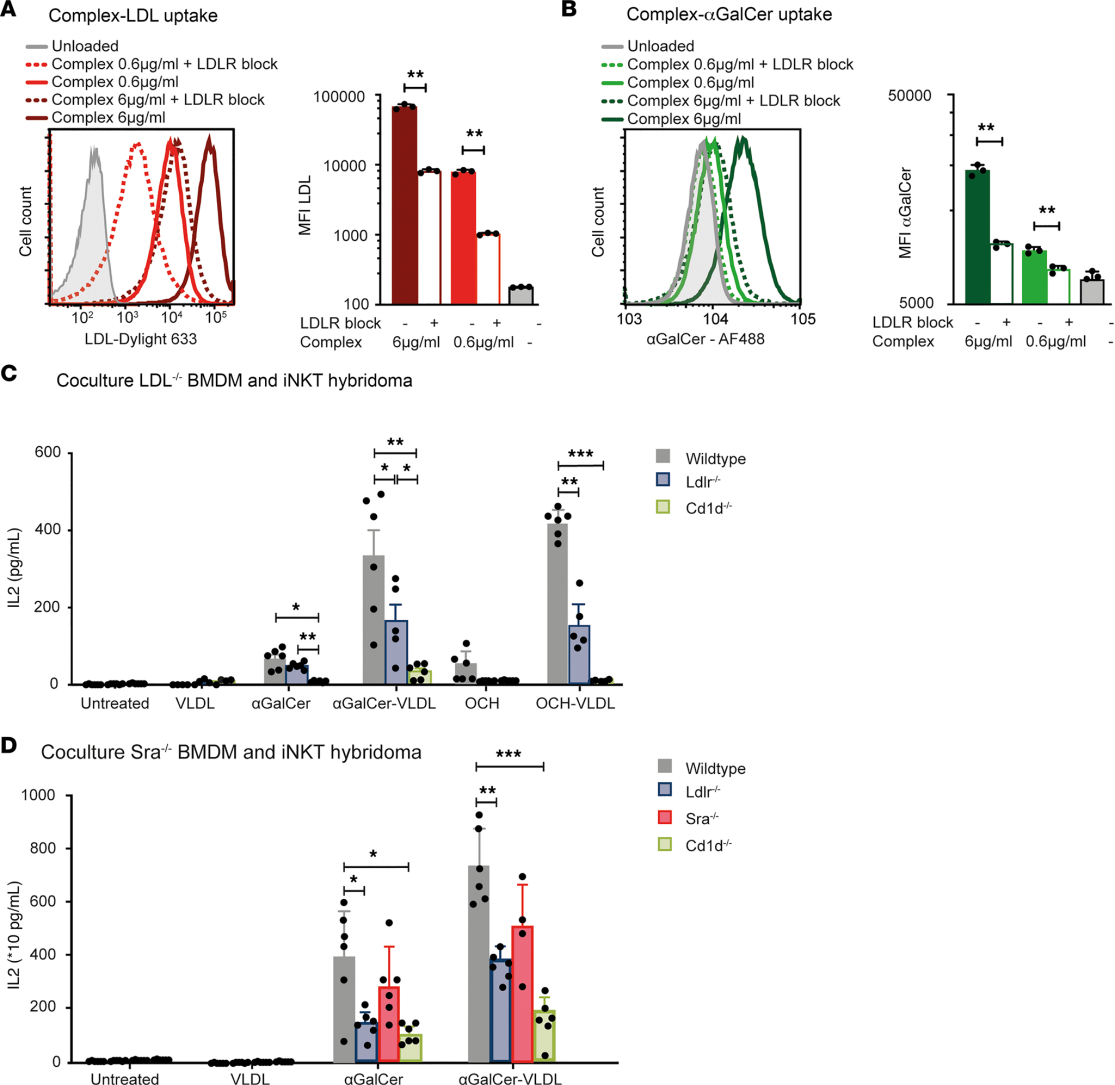
Uptake of lipoprotein-lipid antigen complexes and activation of iNKT cells. (**A** and **B**) Complex uptake upon loading of THP1 cells with different concentrations of fluorescently labeled LDL-αGalCer complexes with or without LDLR-blocking antibody (*n* = 3). (**C**) IL-2 in supernatant coculture of WT, *Ldlr^–/–^*, or *Cd1d^–/–^* mouse BMDMs with DN32.D3-iNKT cells. BMDMs were preloaded with 0.6 μg/mL mouse VLDL-αGalCer complex (10 ng/mL αGalCer), VLDL-OCH complex (100 ng/mL OCH), or equivalent amounts of VLDL, αGalCer, or OCH. Then they were washed and cocultured with DN32.D3 overnight (*n* = 6). (**D**) IL-2 in the supernatant of coculture of WT, *Ldlr^–/–^*, *Sra^–/–^*, or *Cd1d^–/–^* mouse BMDMs with DN32.D3-iNKT hybridoma cells. BMDMs were preloaded with 0.6 μg/mL mouse VLDL-αGalCer complex (10 ng/mL αGalCer) or equivalent amounts of VLDL or αGalCer, washed, and cocultured with DN32.D3 overnight (*n* = 6). Statistics: error bars represent mean ± SD. Unpaired *t* tests (**A** and **B**), 1-way ANOVA with Tukey’s post hoc analysis and multiple-testing correction to compare groups at single time points (**C** and **D**). *P < 0.05; **P < 0.01; ***P < 0.001.

To study the impact of lipoprotein-lipid antigen complexes on iNKT cell activation, we first investigated the uptake of these complexes by APCs, using LDL-αGalCer complexes. Human THP1 monocyte-like cells, which constitutively express LDLR (17), were treated with fluorescently labeled LDL-αGalCer complexes with or without preincubation with LDLR-blocking antibody. We tracked LDLR-mediated uptake of human LDL-αGalCer complexes and found that blocking of the LDLR reduced uptake of both the LDL-DyLight 633 and αGalCer-AF488 complex components in THP1 cells by 80%–90%, showing that uptake of LDL-αGalCer complexes is indeed LDLR dependent (Figure 3, A and B). Next, we studied whether lipoprotein-lipid antigen complexes can activate murine iNKT cells in vitro, to assess functionality of the complexes. For these studies, we used mouse VLDL rather than LDL, to match the recombinant VLDL-αGalCer particles that were generated for the in vivo studies (below), and for practical reasons, as ultracentrifuge-mediated isolation of substantial amounts of mouse VLDL is feasible, while mouse LDL isolation can be complicated (18). Murine WT, *Ldlr^–/–^*, and *Cd1d^–/–^* bone marrow–derived macrophages (BMDMs) were treated with mouse VLDL-αGalCer complexes, VLDL-OCH complexes, or equivalent amounts of VLDL or αGalCer or OCH, then cocultured with mouse DN32.D3-iNKT hybridoma cells. The mouse iNKT TCR is known to have low affinity for iGb3-CD1d complexes and therefore does not elicit a cytokine response in cocultures (19). iNKT cell activation was gauged by measuring IL-2 (Figure 3C) (20), showing robust activation of DN32.D3-iNKT hybridoma cells upon treatment with VLDL-αGalCer and VLDL-OCH complexes. Complex-mediated activation of iNKT cells was CD1d and LDLR dependent (Figure 3C). Nonetheless, the presence of an iNKT cell response in coculture with *Ldlr^–/–^* APCs suggests that the uptake of VLDL-αGalCer complexes by APCs in vivo does not solely depend on LDLR. Previous studies indicated a role for scavenger receptor A (SRA) in the uptake of αGalCer (21). Therefore, we used BMDMs from WT, *Ldlr^–/–^*, *Sra^–/–^*, and *Cd1d^–/–^* mice in a similar experimental set-up, which indicated an additional role for SRA in the uptake of our αGalCer-LDL complexes (Figure 3D). In conclusion, coculture experiments showed potent complex-mediated activation of iNKT cells in vitro in a CD1d- and LDLR-dependent manner. SRA could act as an alternative uptake mechanism for the complexes.

### Lipoprotein-αGalCer complexes activate iNKT cells in vivo in an LDLR-dependent manner.

**Figure 4:**
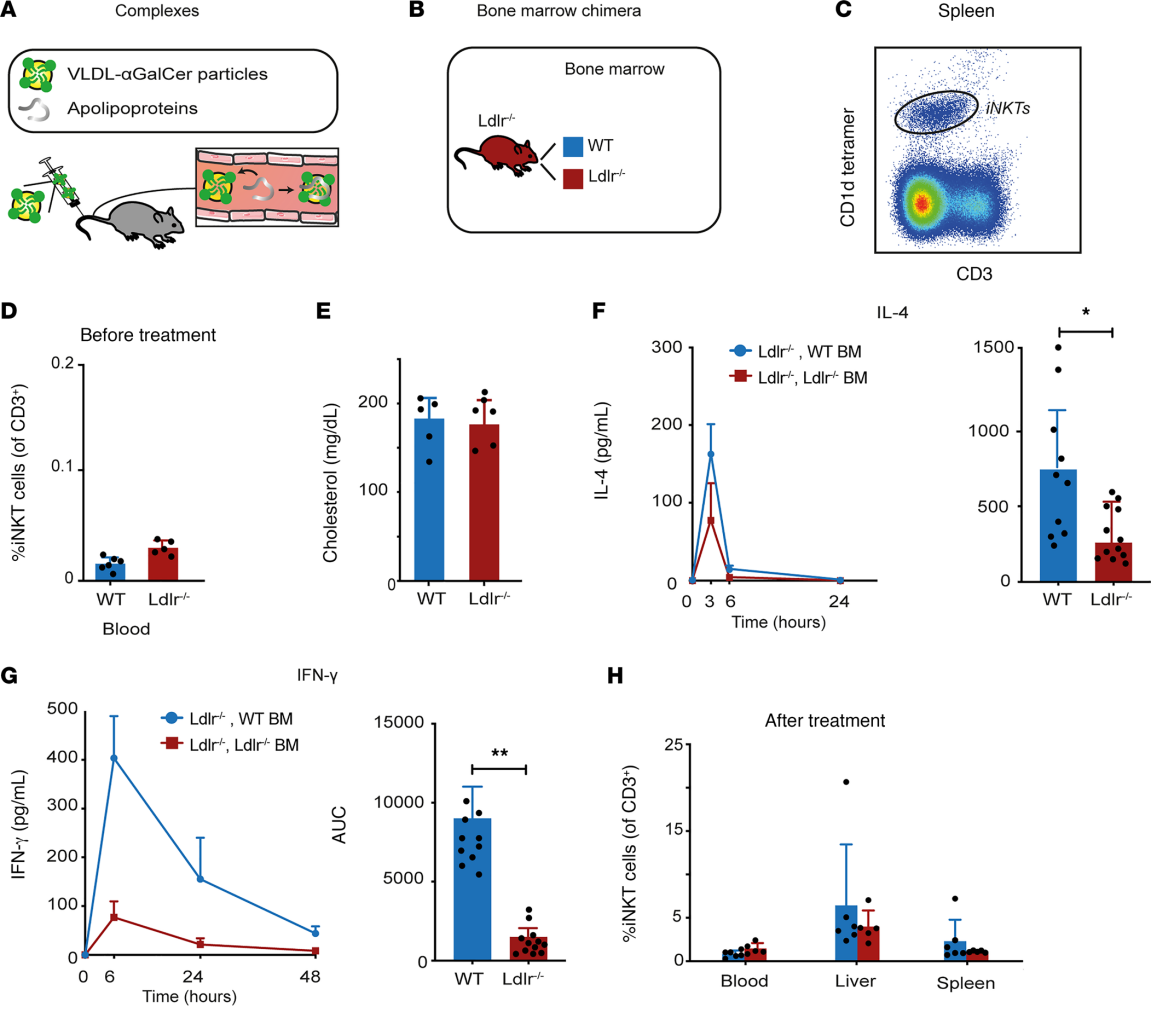
LDLR-proficient APCs promote iNKT cell activation by lipoprotein-αGalCer complexes in hypercholesterolemic mice. (**A**) Recombinant VLDL-αGalCer complexes, free of apolipoproteins, were injected in mice intravenously. (**B**) Bone marrow chimeras of *Ldlr^–/–^* mice with WT or *Ldlr^–/–^* bone marrow were generated. (**C**) Representative flow cytometry image of mouse spleen iNKT cells (*n* = 5–6 mice per group; total 11 mice). (**D**) Percentage of circulating iNKT cells before injection with VLDL-αGalCer particles (*n* = 5–6 mice per group; total 11 mice). (**E**) Serum cholesterol levels (*n* = 5–6 mice per group; total 11 mice). (**F** and **G**) Serum iNKT cell cytokine responses upon injection with VLDL-αGalCer complexes (*n* = 10 *Ldlr^–/–^* WT BM and *n* = 12 *Ldlr^–/–^* *Ldlr^–/–^* BM). (**H**) Percentage of iNKT cells 2 weeks posttreatment (*n* = 5–6 mice per group; total 11 mice). (**D**) Percentage of circulating iNKT cells before injection with VLDL-αGalCer particles (*n* = 5–6 mice per group; total 11 mice). Statistics: error bars represent mean ± SD, except for the cytokine time course in **F** and **G** (mean ± SEM). Unpaired *t* test (**D**–**H**). *P < 0.05; **P < 0.01.

For in vivo experiments we used recombinant protein-free VLDL particles complexed with αGalCer, which only bind to apoE and other exchangeable apolipoproteins in the circulation (Figure 4A) (22). WT mice with LDLR-proficient hepatocytes generally show rapid clearance of VLDL particles, which could limit the uptake by extrahepatic APCs (22). We therefore employed bone marrow chimeras of *Ldlr^–/–^* mice with WT and *Ldlr^–/–^* APCs, to study LDLR-mediated uptake of the VLDL-αGalCer particles (Figure 4B). The chimeras showed similar numbers of APCs, equivalent CD1d expression, and similar circulating iNKT cell numbers pretreatment, while both groups of mice showed characteristic hypercholesterolemia (Figure 4, C–E, and Supplemental Figure 4, A–D). Upon intravenous injection of the recombinant VLDL-αGalCer complexes, both chimeras showed a characteristic iNKT cell response, with circulating IL-4 levels peaking at 3 hours and IFN-γ levels peaking at 6 hours (Figure 4, F and G). *Ldlr^–/–^* mice reconstituted with WT APCs showed the highest IL-4 and IFN-γ levels (Figure 4, F and G). The chimeras did not show differences in iNKT cell numbers and phenotype 2 weeks later (Figure 4H and Supplemental Figure 4, E and F). Taken together, these experiments showed that VLDL-αGalCer complexes activated iNKT cells in vivo. In line with the LDLR-mediated uptake of lipoprotein-αGalCer complexes that we observed in vitro, chimeras with LDLR-proficient APCs showed the highest iNKT cell–associated serum cytokine levels. Nonetheless, the presence of an iNKT cell response in chimeras with *Ldlr^–/–^* APCs suggests that the uptake of VLDL-αGalCer complexes by APCs in vivo does not solely depend on LDLR, in line with our in vitro experiments.

### Human LDLR mutations are associated with impaired iNKT cell proliferation and activation.

**Figure 5:**
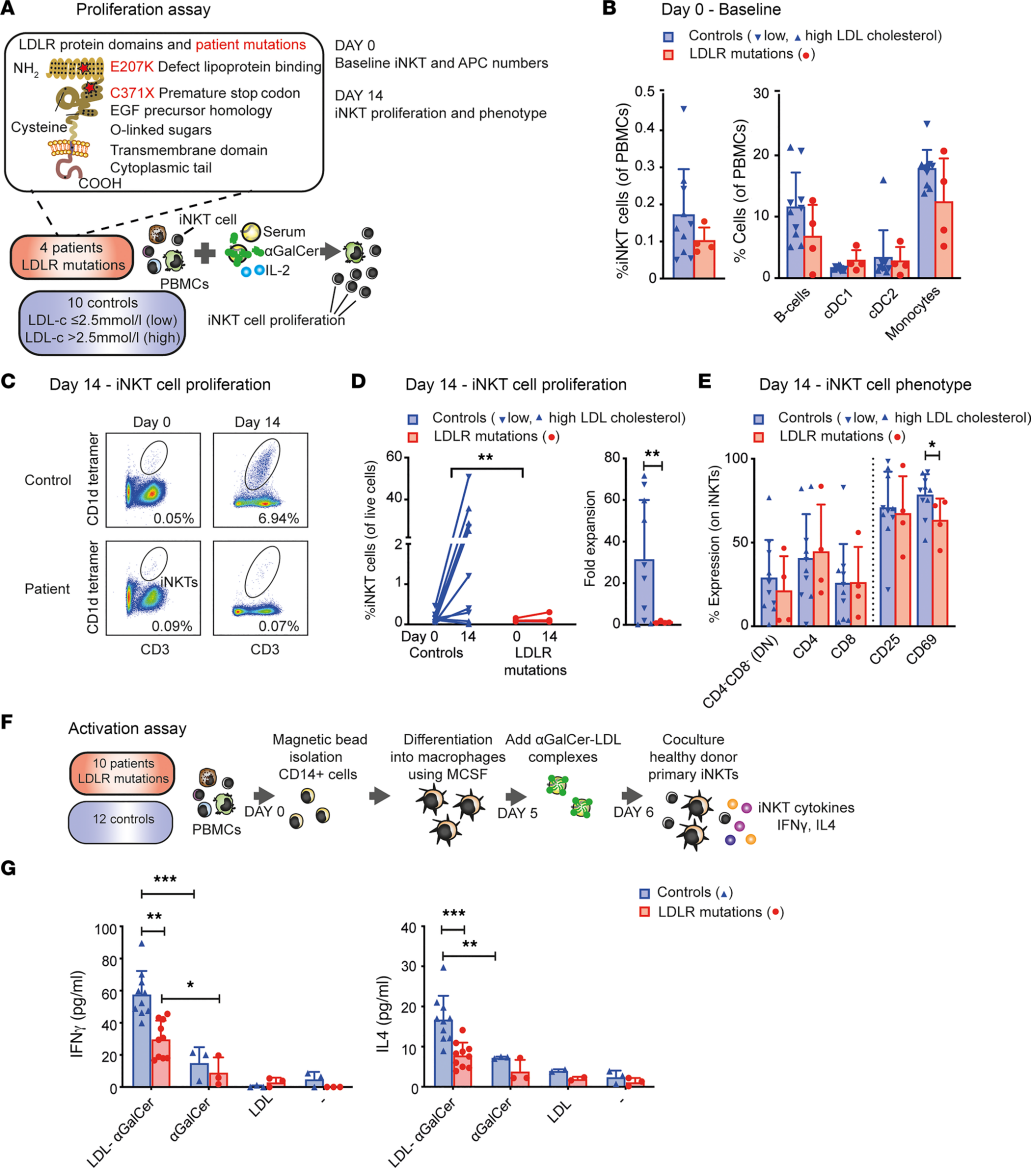
Human LDLR mutations are associated with impaired iNKT cell proliferation and activation. (**A**) Principle of the iNKT cell proliferation assay. PBMCs of patients with LDLR mutations and controls were cultured in presence of lipoproteins, αGalCer, and IL-2 for 14 days. (**B**) Baseline percentage of iNKT cells (left) and APCs, including B cells, cDC1s, cDC2s, and monocytes (right), of LDLR-mutated PBMCs and controls. Blue downward triangles represent controls with low circulating LDL-cholesterol levels (≤2.5 mmol/L, *n* = 5); blue upward triangles represent controls with high circulating LDL-cholesterol levels (>2.5 mmol/L, *n* = 5); and red circles represent LDLR-mutated patients (*n* = 4). (**C**) Representative flow cytometric analysis of LDLR-mutated and control PBMCs on days 0 and 14. Numbers in graphs indicate the percentage of cells in the iNKT cell gate. (**D**) iNKT cell proliferation on day 14 shown as a percentage of iNKT cells of live cells on days 0 and 14 (left) and fold expansion of the iNKT cells after 14 days (right) (*n* = 10 controls, 4 patients). (**E**) Percentage of CD4^–^CD8^–^ (DN), CD4^+^, CD8^+^, CD25^+^, and CD69^+^ iNKT cells (*n* = 10 controls, 4 patients). (**F**) Principle of the iNKT cell activation assay. Monocytes (CD14^+^ cells) were isolated from PBMCs of patients and controls, then cultured in presence of MCSF 5 days for differentiation into macrophages. The medium was changed to low-serum medium (2% FCS), and cells were treated with LDL-αGalCer or equivalent amounts of αGalCer (100 ng/mL) or LDL overnight and washed. Human primary iNKT cells from a healthy donor were added for 24 hours, and cytokine production was measured in the supernatant of the cocultures. (**G**) iNKT cell cytokines in supernatants of LDLR-mutated macrophages and controls in coculture with healthy donor primary iNKT cells. Statistics: error bars represent mean ± SD. Mann-Whitney *U* tests (**D** and **E**), independent *t* test with Bonferroni’s correction for multiple comparisons (**G**). *P < 0.05; **P < 0.01; ***P < 0.001.

To investigate the relevance of lipoproteins as a lipid antigen delivery system in humans, PBMCs from patients with receptor-defective or receptor-negative LDLR mutations and controls were collected from a local cardiovascular biobank (23). First, PBMCs were subjected to a standard iNKT cell proliferation assay using αGalCer as the stimulus in the presence of IL-2, assuming that αGalCer would naturally form complexes with the lipoproteins present in serum (Figure 5A). At baseline, no differences in iNKT cell numbers, APC numbers, or CD1d expression were observed between LDLR-mutant PBMCs and controls (Figure 5B and Supplemental Figure 5A). On day 14, we observed defective iNKT cell proliferation of the LDLR-mutant PBMCs, compared with controls (Figure 5, C and D). The impaired iNKT cell activation and proliferation of LDLR-mutant PBMCs did not coincide with differences in surface marker expression, except for lower expression of the activation marker CD69 (Figure 5E). The impact of the LDLR mutations could not be attributed to high patient cholesterol levels, because PBMCs from controls with high and low circulating cholesterol levels showed similar iNKT cell proliferation (Figure 5D). Second, we assessed the effect of LDLR mutations on complex uptake by APCs and subsequent iNKT cell activation of healthy donor iNKT cells. Monocytes were isolated from PBMCs from patients with LDLR mutations and controls and were cultured with MCSF for 5 days for differentiation into macrophages (*n* = 10 controls and 10 patients). The medium was changed to low-serum medium (2% FCS), which is the minimum amount to ensure viability of the cells for the duration of the experiment. The cells were treated with LDL-αGalCer complexes or equivalent amounts of αGalCer (100 ng/mL) or LDL overnight and subsequently washed (Figure 5F). Human primary iNKT cells from a healthy donor were added to bypass a possible effect of LDLR-deficient iNKT cells. The next day, measurement of iNKT cell cytokines showed significantly reduced secretion of IFN-γ and IL-4 of iNKT cells activated by the LDLR-mutant APCs (Figure 5G). Complex-derived αGalCer elicited a stronger iNKT cell response compared with αGalCer alone, indicating that the bioactivity of complex-derived αGalCer is enhanced. Taken together, human LDLR mutations are associated with impaired iNKT cell proliferation and activation.

## Discussion

In our study, we defined the mechanism of lipid antigen delivery to APCs by circulating lipoproteins. Using 2c-FCCS, we demonstrated stable complex formation of lipid antigens with lipoproteins in vitro and in vivo. Our in vitro experiments showed that the uptake of LDL-lipid antigen complexes by APCs is mediated by LDLR. Subsequently, the lipid antigens are released and loaded onto CD1d, to elicit iNKT cell activation, as illustrated by the potent iNKT cell response in vitro and in vivo. We also demonstrated LDLR-dependent activation of iNKT cells by recombinant VLDL-αGalCer complexes in vivo. Finally, iNKT cells from LDLR-mutant patient PBMCs showed impaired activation and proliferation in response to ligand stimulation, illustrating that LDLR-mediated uptake of lipoprotein-lipid antigen complexes is also relevant for iNKT cell activation in humans. Taken together, our study shows that lipoproteins can act as vehicles for lipid antigen delivery and iNKT cell activation in mice and humans and provides insight into the immunological role of lipoproteins.

Many established iNKT cell antigens have a ceramide backbone (sphingolipids composed of N-acetylsphingosine and fatty acid), and our findings are in line with the established biochemical properties of ceramides (1, 24, 25). Biochemical studies have shown that 98% of circulating ceramides are present in the serum lipoprotein compartment. Moreover, various glycosylceramides can insert into phospholipid rafts, which are structurally similar to the phospholipid shell of lipoproteins (9, 10). Finally, previous studies have shown that apoE facilitates the uptake of αGalCer, αGalGalCer, and even CD1b- and CD1c-associated lipid antigens such as glucose monomycolate, mycolic acid, and manosyl-phosphomycoketide (3, 4). In a concerted fashion, apolipoproteins and their associated lipoproteins may thus be involved in transport and uptake of a broader range of CD1-associated antigens. Taken together, lipoproteins likely act as a vehicle for delivery of lipid antigens with a ceramide backbone and possibly a broader range of CD1-associated antigens.

The biochemical characteristics of the lipoprotein phospholipid shells may also explain the lack of complex formation we observed for HDL and αGalCer. The phospholipid shell of HDL has a high apolipoprotein content, which decreases lipid fluidity and diffusion of glycosylceramides, and may similarly impair αGalCer binding and insertion (9, 26). In our functional studies, we have therefore focused on LDL and VLDL, rather than HDL. Notably, lack of complex formation in vitro does not necessarily imply that HDL cannot transport foreign lipid antigens in vivo. Although the vast majority of lipid antigens were found in the LDL fraction of human serum, we found small amounts of lipid antigens in the HDL fraction of human serum spiked with lipid antigens (Figure 2D). Circulating phospholipid transfer protein and cholesteryl ester transfer protein have been implicated in transfer of ceramides between human lipoprotein classes (8, 25, 27), suggesting that active transfer of αGalCer and other lipid antigens from VLDL or LDL onto HDL may occur. In other words, we cannot preclude a minor role for HDL in lipid antigen delivery in vivo.

The uptake of lipoprotein-αGalCer complexes by APCs is LDLR mediated. Our in vitro studies showed 80%–90% reduction of lipoprotein-αGalCer uptake by THP1 monocytes upon LDLR blocking, and impaired iNKT cell activation in cocultures with LDLR-deficient BMDMs, compared with WT BMDMs (Figure 3C). The modest iNKT cell response in chimeras with *Ldlr^–/–^* APCs and the modest iNKT cell response to *Ldlr^–/–^* BMDMs in vitro nonetheless suggest that other lipoprotein receptors can also contribute to the uptake of VLDL-αGalCer complexes. Previous studies indicated a role for SRA in the uptake of αGalCer, the prototypic lipid antigen for iNKT cell activation (21). Therefore, we assessed the role of SRA in uptake of lipoprotein-lipid antigen complexes in vitro and showed that SRA provides an alternative route for uptake of lipoprotein-αGalCer complexes (Figure 3D). This uptake mechanism could explain the remaining iNKT cell activation elicited by *Ldlr^–/–^* APCs in vitro and in vivo. Other alternative uptake mechanisms for lipoprotein-lipid antigen complexes may include sortilin and other members of the LDLR family, such as LRP1 (11, 28). In case of LDLR dysfunction or lipoprotein deprivation, cells depend on these alternative uptake mechanisms, next to intracellular cholesterol synthesis, to meet their cholesterol requirements (28, 29). LDLR-mediated uptake of lipoprotein-ligand complexes could have additional benefits for antigen presentation, as complex-derived αGalCer and OCH showed greater iNKT cell activation than αGalCer alone (Figure 3C). This is in line with previous studies showing fast LDLR-mediated endocytosis and endolysosomal traversal of lipoprotein-containing cargo, which may accelerate lipid antigen loading and presentation by APCs by directing the cargo to relevant intracellular compartments enriched in CD1d molecules (20, 30).

Finally, the discovery of lipoproteins as a lipid antigen delivery system has translational implications. First, our study showed that complex formation of lipid antigens with lipoproteins enhances the bioactivity of lipid antigens, deepening our understanding of the consequences of iNKT cell activation in health and disease. The pathophysiology of atherosclerosis is particularly intriguing, as the interplay between lipoproteins and the immune system has proved key in its development (31). Lipoprotein particles enter the subendothelial space, mainly at sites of low shear stress due to hemodynamics, and undergo modification. Modified lipoproteins, such as oxidized LDL, activate the immune system in the subendothelial space by acting as adjuvant molecular patterns, triggering an innate immune response (32, 33), and by acting as antigens, stimulating an adaptive immune response (34, 35). Our study defines a potentially novel role for lipoproteins in iNKT cell activation and furthers our understanding of the interplay between lipoproteins and the immune system, with implications for the activation of iNKT cells in lipoprotein-rich environments such as the subendothelial space, where atherosclerotic plaques develop (36–38). Second, our findings call into question the use of *ApoE^–/–^* and *Ldlr^–/–^* mouse models to study the role of iNKT cells in atherosclerosis and other diseases. Mouse models with an intact apoE/LDLR pathway for antigen uptake and atherogenic lipid clearance would be preferred, as discussed elsewhere (39). Third, lipoprotein uptake is determined by lipoprotein receptor expression profiles, which may be harnessed for specific treatments. iNKT cells, for example, display direct cytotoxic effects on tumor cells expressing CD1d, and administration of αGalCer as an adjuvant in cancer therapy has shown promising results in mice (40–43). Hematopoietic cells, especially those of myelomonocytic and B cell lineages, express high levels of both CD1d and LDLR (30, 44, 45). Administration of lipoprotein-αGalCer complexes might therefore fuel the potency of αGalCer as an adjuvant in treatment of hematopoietic cancers. Finally, lipoproteins not only transport lipid antigens but also have been implicated in transport and LDLR-mediated uptake of lipopolysaccharides and other bacterial lipids (46, 47). The emergence of lipoproteins as a delivery system may not have broader implications, as other circulating lipid-based immune mediators might also avail themselves of this delivery route.

## Methods

### Patient samples

PBMC samples of 14 patients with LDLR mutations and 20 age-matched controls were provided by the Ucorbio Cardiovascular Biobank of the University Medical Center in Utrecht, the Netherlands (ClinicalTrials.gov NCT02304744).

#### Proliferation assay.

Patients included 3 men and 1 woman, mean age 58 years, with circulating LDL-cholesterol levels 1.4–6 mmol/L and LDLR mutations C371X or E207K. Controls included 6 men and 4 women, mean age 60 years, of whom 5 had normal circulating LDL-cholesterol levels (<2.6 mmol/L) and 5 had high circulating LDL-cholesterol levels (≥2.6 mmol/L).

#### Cytokine assay.

Patients included 3 men and 7 women, mean age 58 years, with LDLR mutations Padova-2, N543H, 191-2, E207K, R612H, D206E, and 313+1/2 and average LDL-cholesterol level 5.4 mmol/L. Controls included 2 men and 8 women, mean age 55 years, with average LDL-cholesterol level 2.3 mmol/L.

### Animal studies

Male *Ldlr^–/–^* mice on a C57BL/6 background and male C57BL/6 control mice (WT) were purchased from The Jackson Laboratory, bred in-house under specific pathogen–free conditions, and fed a standard chow diet. To create bone marrow chimeras, 10- to 12-week-old CD45.2 *Ldlr^–/–^* animals underwent irradiation and transfer of either CD45.1 *Ldlr^–/–^* or WT bone marrow cells. At 18–20 weeks of age, circulating APC and iNKT cell origin was assessed (>98% donor origin, Supplemental Figure 3B). For the in vivo challenge with lipoprotein-αGalCer complexes, mice received an intravenous injection of VLDL-like particles containing 1 mg of triglycerides and 5 μg of αGalCer (22), followed by repetitive blood sampling. The iNKT cell response cytokines IL-4 and IFN-γ were measured in serum using ELISA (BioLegend). Two weeks later, mice were sacrificed, and blood, spleen, and liver were harvested for further analysis of iNKT cell numbers and phenotype using flow cytometry.

### Isolation of mouse leukocytes and flow cytometry

#### Blood leukocytes.

Upon removal of serum for total cholesterol measurement (Randox), blood samples were treated with red blood cell lysis buffer (MilliporeSigma) and washed using RPMI including 2% FCS and antibiotics (Gibco, Thermo Fisher Scientific).

#### Splenic leukocytes.

Spleens were minced through a 70 μm mesh filter (Falcon, Corning), collected in red blood cell lysis buffer, and washed.

#### Liver leukocytes.

Livers were dissected into small pieces and dissociated using collagenase IV (MilliporeSigma C5138) and DNase I (MilliporeSigma/Roche) using an established protocol (48). Subsequently, dissociated livers were minced over a 70 μm mesh filter, and liver leukocytes were harvested using Ficoll-Hypaque density centrifugation (Histopaque, MilliporeSigma), then washed.

#### Flow cytometry.

Mouse leukocytes were dissolved in FACS buffer (2% FCS, 2 mmol/L EDTA, and 0.1% NaN_3_ in PBS), preincubated with 10% rat serum in FACS buffer to block nonspecific binding, and stained with a viability dye (BioLegend) and antibodies specific for CD3 (145-2C11), CD4 (RM4-5), CD8 (53-6.7), CD25 (PC61), CD45.1 (A20), CD45.2 (30-F11), NK1.1 (PK136), and CD1d tetramer (NIH) for iNKT cell phenotyping and antibodies specific for CD1d (1B1), CD11b (M1/70), CD11c (N418), CD19 (1D3), CD45.1 (A20), CD45.2 (30-F11), CD64 (X54-5/7.1), F4/80 (BM8), Ly6C (EPR27220-67), and MHCII (M5/114.15.2) for APC phenotyping (from BioLegend, except for the CD1d tetramer). Cells were analyzed using an LSRFortessa flow cytometer (BD Biosciences) with FACSDiva software (BD Biosciences), and data were analyzed with FlowJo software v10 (Tree Star Inc).

### Synthesis of AF488-labeled αGalCer

AF488-labeled αGalCer was synthesized following reported procedures (49). The fluorescent label was introduced at the α-position of the acyl chain of αGalCer via click chemistry. First, AF488-PEG4-alkyne was synthesized by stirring a solution of AF488 NHS Ester (Thermo Fisher Scientific A20000) (1.0 mg, 1.6 μmol) and amino-PEG4-alkyne (Sigma-Aldrich 764248) (0.4 mg, 1.6 μmol) in N,N-dimethylformamide (DMF; 0.5 mL, MilliporeSigma) overnight. Concentration of the reaction mixture under reduced pressure, followed by trituration with diethyl ether (2 × 0.5 mL), afforded AF488-PEG4-alkyne in quantitative yield. Second, CuSO_4_ solution (6 μL of a 0.5 M solution, 3 μmol) and a sodium ascorbate solution (13 μL of a 1.0 M solution, 13 μmol) were added to a solution of the 2-azide derivative of αGalCer (1.3 mg, 1.6 μmol) and the AF488-PEG4-alkyne (1.2 mg, 1.6 μmol) in a t-BuOH/H_2_O mixture (0.5 mL, 1:1) at room temperature. The reaction mixture was heated for 10 hours at 50°C, diluted with CHCl_3_ (5 mL), and washed with brine (1.5 mL). The phases were separated, and the aqueous layer was extracted with CHCl_3_ (2 × 2.5 mL). The combined organic layers were dried over Na_2_SO_4_, and the volatiles were removed under reduced pressure. Purification of the residue by flash column chromatography (CHCl_3_/MeOH/H_2_O, 65:25:4) afforded AF488-labeled αGalCer. Quality control of the AF488-labeled αGalCer showed similar ligand activity as unlabeled αGalCer (Avanti Lipids) in standard iNKT cell activation assays (not shown).

### Fluorescence labeling of iGb3 and OCH

iGb3 and OCH were fluorescently labeled using a DyLight 488 NHS Ester (Thermo Fisher Scientific) as labeling via click chemistry was not available for these lipid antigens. The NHS Ester and DMF were first equilibrated to room temperature. Then, 50 μg of DyLight 488 was dissolved in 50 μL of DMF (1 mg/mL), was vortexed, sat for 5 minutes at room temperature, and was vortexed again. The lipid antigen, triethylamine, and NHS Ester were mixed in a 1.3:1.0:1.0 ratio and incubated for 1 hour at room temperature on a shaker protected from light. The labeled lipid antigens were stored at 4°C protected from light for up to 48 hours.

### Lipoprotein-lipid antigen complex formation

Lipoproteins were isolated from human serum (NHS blood donation service) and mouse serum using established ultracentrifugation protocols (105,000*g* for 20 hours with brakes off) (50). For 2c-FCCS and imaging studies, isolated lipoprotein fractions were fluorescently labeled using a D)yLight 633 NHS Ester (Thermo Fisher Scientific). For in vitro iNKT cell activation assays, unlabeled lipoprotein fractions and unlabeled lipid antigens were used. For complex formation, lipoproteins were incubated with lipid antigens in a 1:10 molar ratio at 37°C on a shaker for 1 hour. For example, 1 mL of human LDL (apolipoprotein content 2 mg/mL, approximately 4 μM) was incubated with 34 μL αGalCer (1 mg/mL in DMSO or sucrose/l-histidine/Tween, approximately 40 μM).

### 2c-FCCS

DyLight 633–labeled lipoproteins, AF488-labeled αGalCer, DyLight 488–labeled OCH, DyLight 488–labeled iGb3, and lipoprotein-lipid antigen complexes were 200× diluted in PBS with 0.1% FCS, then separately loaded on 8-well chambered coverslips (Ibidi). For in vivo complexation, 200 μL of DyLight 633–labeled human LDL and AF488-labeled αGalCer were mixed in a 1:10 molar ratio and instantly injected intravenously in an *Ldlr^–/–^* mouse. After 30 minutes, serum was harvested and 20× diluted in PBS, then loaded on the chambered coverslip. Samples were warmed to 37°C before measurement on a ZEISS LSM880 Inverted Confocal Microscope System, using a designated 2c-FCCS Plan Apochromat 40×/1.2 NA water objective and Zen Black 2.1 SP3 LSM software with 2c-FCCS module (ZEISS). Laser alignment and pinhole position were optimized for 2c-FCCS, using a 488/633 laser power of ≈3–4 μW set to prevent photobleaching. All samples were measured in 3 runs of 6 repetitive measurements of 10 seconds. Upon exclusion of measurements with aggregates, 15 representative correlation measurements G(τ) per sample underwent curve fitting to extract diffusion times (τ_D_) and counts per molecule, using a model for free diffusion in 3D with 1 diffusive species and a triplet state with a fixed decay time τ_F_ = 5 μs in the Zen software FCS module (ZEISS).


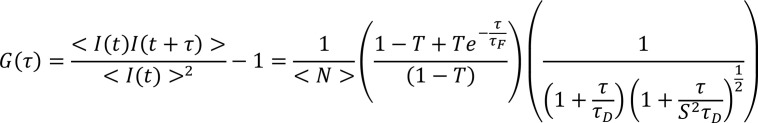
Equation 1

where <I(t)> is the mean intensity in the focal volume, <N> is the average number of molecules in the focal volume, T is the relative fraction of the triplet state, and S = ω_z_/ ω_xy_ is the ratio of axial to radial (1/e^–2^) radii of the measurement volume. For our measurements we set S = 5. The counts per molecule were calculated from <I(t)>/<N>.

### Sample preparation and LC-MS sphingolipidome analysis

Organic solvents were UPLC-MS grade and purchased from J.T. Baker. Chemicals were analytical grade and purchased from Sigma-Aldrich. Glycosphingolipid standards 17:0 iGB3 and αGalCer C26 were obtained from Avanti Polar Lipids (Sigma-Aldrich). The standard OCH was obtained from the NIH Tetramer Core Facility. Water was obtained fresh from a Milli-Q instrument (Merck Millipore).

Samples were transferred to 2 mL Eppendorf safe-lock tubes and evaporated to dryness in a Labconco Centrivap (VWR). To the residue, 100 μL water, 5 μL Cer/Sph Mixture 1 internal standard, 500 μL methanol, and 250 μL chloroform were added. After pulse vortex mixing, the samples were incubated overnight in a VWR thermostated shaker (~17 hours, 900 rpm, 48°C). After allowing the samples to cool to room temperature, a volume of 75 μL 1 M KOH was added to each sample. Samples were vortex mixed and incubated for 2 hours (900 rpm, 37°C). After centrifugation at room temperature (5 minutes, 15,000*g*), a volume of 100 μL was transferred to an injection vial for LC-MS analysis.

The LC-MS sphingolipidome analysis was performed using a 2.1 × 50 mm Acquity BEH C18 UPLC column (Waters) installed into an UltiMate 3000 LC system (Thermo Fisher Scientific). Samples were kept at 10°C in the autosampler. The column outlet was coupled to a Thermo Fisher Scientific Q Exactive FT mass spectrometer equipped with an electrospray ion source. The UPLC system was operated at a flow rate of 300 μL/min, and the column was kept at 60°C. The mobile phases consisted of 24% (v/v) methanol, 25% (v/v) acetonitrile, 20% (v/v) isopropanol, 30% (v/v) water, 1% (v/v) formic acid, and 10 mM ammonium formate (A) and 69% (v/v) methanol, 20% (v/v) isopropanol, 10% (v/v) acetonitrile, 1% (v/v) formic acid, and 10 mM ammonium formate (B), respectively. An 8-minute linear gradient of 0%–100%B was started after the injection of 5 μL of sample, after which the system was kept at 100%B for 2 minutes followed by 6 minutes of column regeneration at 0%B. MS data were acquired in data-directed tandem MS mode over a scan range of *m/z* 200 to 1,200. The system was operated at 120,000 mass resolution in positive mode (+2.5 kV). High mass resolution mass calibration was performed before each experiment. Further MS settings were automatic gain control target: 1 × 10^6^; maximum imaging time: 100 ms; capillary temperature: 300°C; sheath gas: 35 AU; auxiliary gas: 2 AU; spare gas: 0 AU; S-lens radio frequency level: 65. Raw data files were analyzed using Thermo Xcalibur Processing and Quan software.

### Uptake of lipoprotein-αGalCer complexes

THP1 cells (American Type Culture Collection), known for their constitutive LDLR expression (17), were cultured in RPMI 1640 (Gibco, Thermo Fisher Scientific) supplemented with 10% heat-inactivated FCS (ultra-low endotoxin, Biosera), 4 mmol/L Glutamax (Gibco, Thermo Fisher Scientific), 5 mmol/L sodium pyruvate (MilliporeSigma), and antibiotics (Gibco, Thermo Fisher Scientific). For the uptake experiments, THP1 cells were washed in PBS, and 100,000 THP1 cells were seeded in 100 μL Opti-MEM (Gibco, Thermo Fisher Scientific) on a 96-well plate (Corning). Upon preincubation with 5 μg/mL LDLR-blocking antibody for 30 minutes (AF2148, R&D Systems, Bio-Techne), THP1 cells were incubated for 4 hours with 0.3 μL (6 μg/mL) or 0.03 μL (0.6 μg/mL) lipoprotein-αGalCer complexes per well, respectively, containing 100 ng and 10 ng αGalCer. Finally, loading of the DyLight 633–lipoprotein and AF488-αGalCer was assessed on a FACSCanto II flow cytometer (BD Biosciences) with FACSDiva software (BD Biosciences), and data were analyzed with FlowJo software v10.

### Loading of lipoprotein-αGalCer complexes for colocalization analysis

#### Macrophage differentiation.

Human PBMCs were isolated from a single blood transfusion donor, using Ficoll-Hypaque density centrifugation of leukocytes from a leukoreduction system chamber (NHS blood transfusion service). Subsequently, lymphocytes and monocytes were separated in a JE6 elutriator (Beckman Coulter), and monocytes were differentiated into macrophages on glass coverslips (SLS) in 5 days, using RPMI 1640 medium containing 100 ng/mL human MCSF (Miltenyi Biotec), next to 10% heat-inactivated FCS, 4 mmol/L Glutamax, 5 mmol/L sodium pyruvate, and antibiotics (Gibco, Thermo Fisher Scientific).

#### Complex loading studies.

Macrophages were washed with PBS, then incubated for 1 hour at 4°C with 25 μL/mL lipoprotein-αGalCer complexes in Opti-MEM (containing 800 ng/mL αGalCer), following established lipoprotein-loading protocols (50). Upon complex loading, cells were washed with cold PBS, fixed in 4% paraformaldehyde (Thermo Fisher Scientific) for 15 minutes at 4°C, and stored in PBS at 4°C in the dark until further analysis.

#### Confocal microscopy.

Colocalization was assessed using a Nikon A1R HD25 inverted confocal microscope with a Plan Apo 60×/NA 1.40 objective and Galvano scanning mode, and the adjoining NIS-Elements imaging software. Standardized Nyquist sampling of 3 separate planes in 5 representative cells was performed (*n* = 15). Data were analyzed in ImageJ (NIH) using the Plot Profile and JACoP plugin and analysis protocol (51).

### In vitro iNKT cell assays

#### Mouse coculture assay.

Bone marrow cells of C57BL/6, *Ldlr^–/–^*, and *Cd1d^–/–^* mice were differentiated into BMDMs over 5 days using 100 ng/mL murine MCSF (PeproTech, Thermo Fisher Scientific). Upon differentiation, BMDMs were loaded for 4 hours with 0.6 μg/mL mouse VLDL, 0.6 μg/mL VLDL-αGalCer complex (containing 10 ng/mL αGalCer), or αGalCer (10 ng/mL) in Opti-MEM, then washed with PBS. Next, 1.5 × 10^5^ BMDMs per well were cocultured with 1.5 × 10^5^ mouse DN32.D3 iNKT hybridoma cells for 16 hours in culture medium. Coculture supernatants were harvested and analyzed for mouse IL-2 levels using ELISA (R&D Systems, Bio-Techne).

#### Human iNKT cell proliferation assay.

PBMCs of patients with LDLR mutations and age-matched controls were subjected to a standard proliferation assay (52). In short, PBMCs were incubated with 10% heat-inactivated human AB serum, 100 ng/mL αGalCer, and 100 IU/mL human IL-2 (R&D Systems, Bio-Techne) in standard T cell medium. On day 0, baseline APC and iNKT cell phenotypes were assessed using flow cytometry. On day 14, iNKT cell proliferation and phenotype were assessed using flow cytometry. Cells were stained with a viability dye (BioLegend) and mAbs specific for CD3 (HIT3a), CD4 (L200), CD8 (RPA-T8), CD25 (2A3), CD69 (FN50), and CD1d tetramer (NIH) for iNKT cell phenotyping (from BioLegend, except for CD1d).

#### Human iNKT cell activation assay.

Monocytes (CD14^+^ cells) were isolated from PBMCs of patients and controls using magnetic bead separation (Miltenyi Biotec). The CD14^+^ cells were cultured in a flat-bottom, 96-well plate for 5 days in the presence of MCSF for differentiation into macrophages. The medium was changed to low-serum medium (2% FCS), and cells were treated with LDL-αGalCer or equivalent amounts of αGalCer (100 ng/mL) or LDL overnight and subsequently washed. A primary human iNKT cell line was generated from a healthy donor using established protocols (52). In short, blood cones from healthy donors were purchased from the NHS blood service at the John Radcliffe Hospital in Oxford. PBMCs were isolated using Histopaque (MilliporeSigma 1077) centrifugation at 400*g*, for 30 minutes, at room temperature. Lymphocytes were subsequently isolated from PBMCs by counter-current centrifugal elutriation. Next, iNKT cells were separated by bead-based cell separation (Miltenyi Biotec) according to the manufacturer’s instructions. Then, 1 × 10^6^ cells/mL iNKT cells were cultured in a 1:1 ratio in a 24-well plate with feeder cells in RPMI 1640 supplemented with 10% heat-inactivated FBS, 1% penicillin/streptomycin, 15 mM Glutamax, 50 μM 2-ME including 100 ng/mL αGalCer, and IL-2 (2 × 10^6^ IU/mL). After 2 weeks, 50% of the medium was replaced every 3–7 days in fresh medium including IL-2 (100 IU/mL). Cells were split in a 1:2 ratio in the event of confluence of the well. The iNKT cells were frozen in our liquid nitrogen facility, then thawed at the time of the experiment. Purity of the primary cell lines were determined by CD1d tetramer staining using flow cytometry. Cell lines with more than 60% purity were used in coculture assays. Human primary iNKT cells from a healthy donor were added for 24 hours in a 1:1 ratio. A small supernatant fraction was harvested to measure the iNKT cell cytokines IL-4 and IFN-γ using a multiplex immunoassay (Luminex).

### Statistics

Data are presented as mean ± SD, unless otherwise indicated. The Shapiro-Wilk test was used to assess normality of the data. The null hypothesis (H_0_) was that the data were normally distributed. Variables with a Shapiro-Wilk test with *P* > 0.05 were considered normally distributed. Statistical significance between 2 groups was determined using 2-tailed unpaired *t* tests for parametric data, unequal variances *t* tests for parametric data with unequal variances between groups, and Mann-Whitney *U* tests for nonparametric data. Bonferroni’s correction was used to correct for multiple comparisons. To analyze the means of multiple groups involving 1 independent variable, 1-way ANOVA testing was used. The Tukey post hoc test was used to run pairwise comparisons among each of the groups. A *P* value less than 0.05 was considered significant. Statistical analyses were performed using Prism 8 for MacOS (GraphPad Software).

### Study approval

Animal studies were performed according to UK Home Office regulations and European Directive 2010/63/EU and approved by the University of Oxford Animal Welfare Ethical Review Board in Oxford, United Kingdom. Human studies were approved by the Medical Research Ethics Committee of the University Medical Center Utrecht (protocol numbers 11-183 and 19-081). Written informed consent was obtained from all participants.

## Author contributions

SEE, FAV, BCL, LIEY, AM, and HSS performed experiments and data analysis. CMCG and ECAS helped with the mass spectrometry studies. MG and YXZ helped with the animal studies. SK and PCNR provided recombinant VLDL-αGalCer complexes. PJJ and GSB synthesized AF488-labeled αGalCer. JMK, GKH, SH, and FWA facilitated the use of LDLR-mutated PBMCs. HS designed and supervised the study. SEE and HSS wrote the manuscript, and SEE, FAV, AM, BCL, JMK, LIEY, YXZ, CMCG, SK, MG, RJVE, PJJ, GSB, SH, FWA, EK, HLP, MB, VC, GKH, MS, ECAS, PCNR, CM, and HSS reviewed and approved the final manuscript.

## Supplementary Material

Supplemental data

## Version 1 03/28/2023

In-Press Preview

## Version 2 05/08/2023

Electronic publication
