# Supplementary material for: Lipoproteins act as vehicles for lipid antigen delivery and activation of invariant natural killer T cells
Source: JCI Insight. 2023 May 8;8(9):e158089. doi: 10.1172/jci.insight.158089 (PMC10243825; doi:10.1172/jci.insight.158089)
Supplement: Supplemental data [file jciinsight-8-158089-s060.pdf]

Lipoproteins act as vehicles for lipid antigen delivery and activation of invariant Natural Killer T-cells

Suzanne E. Engelen[1], Francesca A. Ververs[2], Angela Markovska[2], B. Christoffer Lagerholm[3], Jordan M. Kraaijenhof[4], Laura IE Yousif[1], Yasemin-Xiomara Zurke[1], Can M.C. Gulersonmez[5], Sander Kooijman[6], Michael Goddard[1], Robert J. van Eijkeren[5], Peter J. Jervis[7], Gurdyal S. Besra[7], Saskia Haitjema[8], Folkert W. Asselbergs[9,10], Eric Kalkhoven[5], Hidde L. Ploegh[11], Marianne Boes[2], Vincenzo Cerundolo[12,13], G.K. Hovingh[4], Mariolina Salio[12], Edwin C.A. Stigter[5], Patrick C.N. Rensen[6],

Claudia Monaco[1], Henk S. Schipper[1,2,14]*

SUPPLEMENTARY FIGURES

Supplemental fig 1. Complex formation lipoproteins - αGalCer

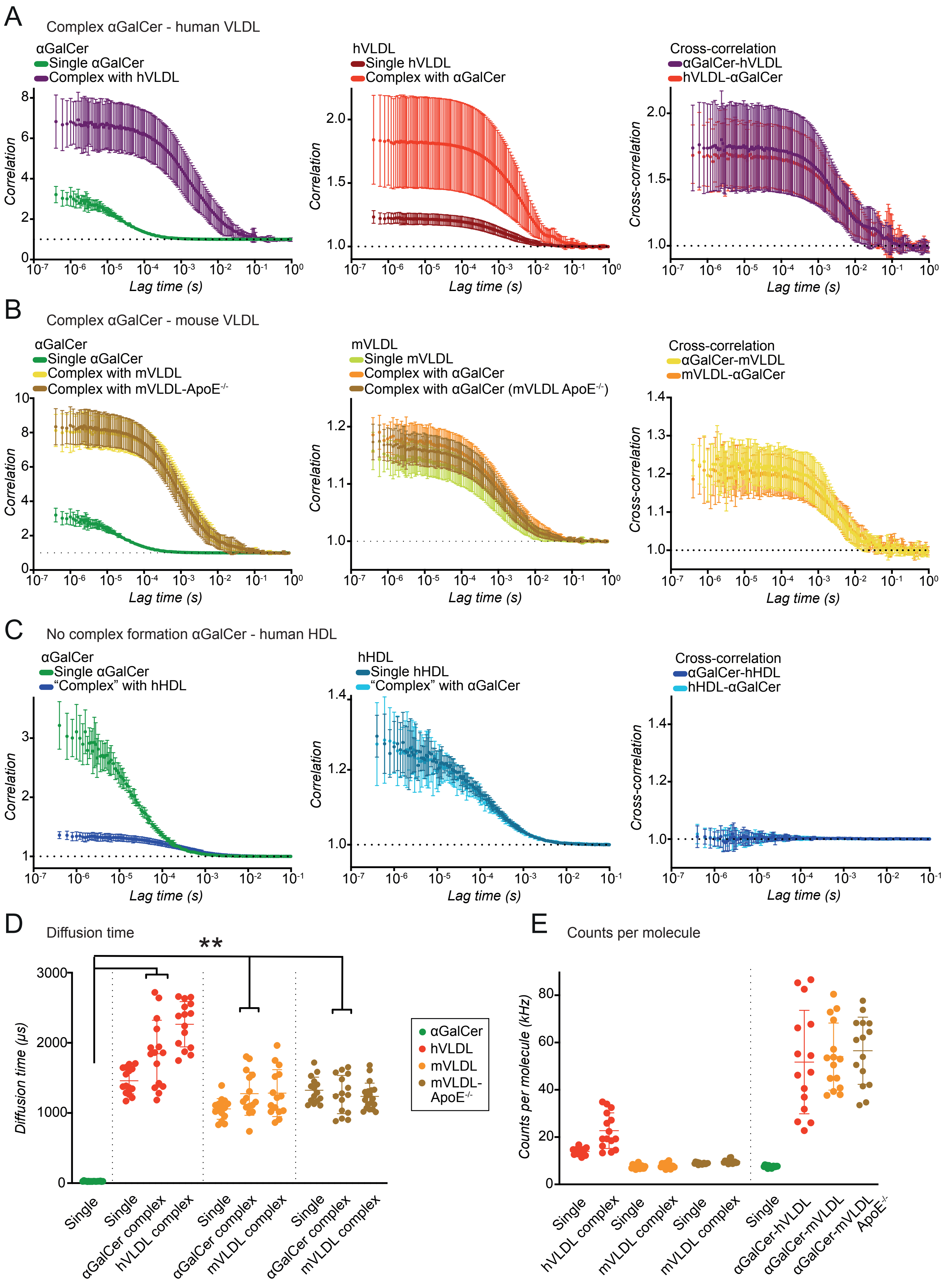

Supplemental figure 1. Complex formation of lipoproteins and αGalCer.
(A) Autocorrelation and cross-correlation curves of αGalCer-AF488 and Dylight-633 labeled human VLDL, comparing single components and complexes. (n = 15). (B) Autocorrelation and cross-correlation curves of αGalCer-AF488, Dylight-633 labeled mouse VLDL, and ApoE$^{-/-}$ mouse VLDL. (n = 15). (C) Autocorrelation and cross-correlation curves of αGalCer-AF488 and Dylight-633 labeled human HDL. The autocorrelation curves do not show synchronization of αGalCer with HDL, neither does the cross-correlation. (n = 15). (D) Diffusion times of αGalCer, human VLDL and mouse VLDL, both as single components and in complex. (n = 15). (E) Fluorescent counts per molecule of single αGalCer, human VLDL and mouse VLDL, both as single components and in complex. (n = 15). Statistics: error bars represent mean ± SD. Unpaired t tests (panel D-E). * $P < 0.05$; ** $P < 0.01$.

Supplemental fig 2. Lipoprotein-lipid antigen complex stability

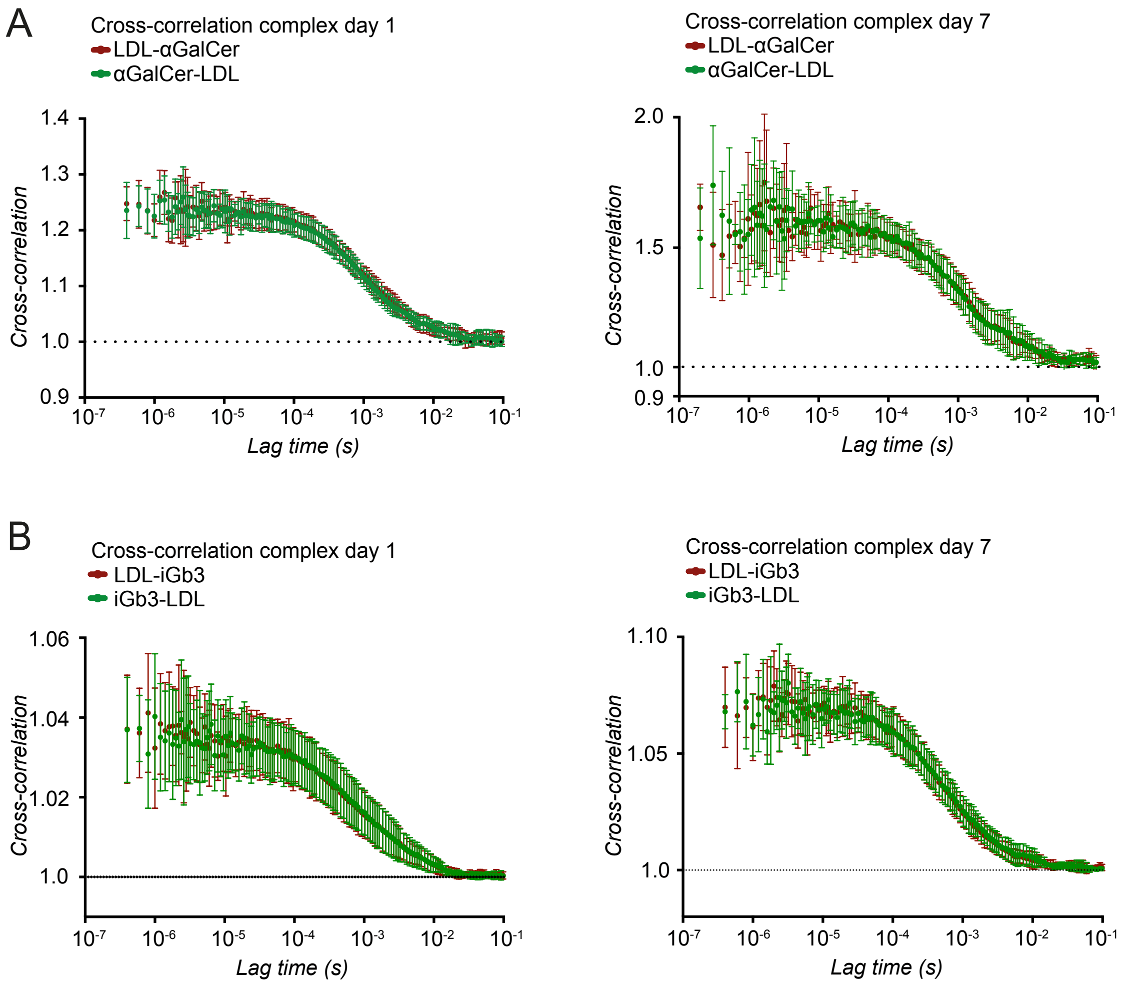

Supplemental figure 2.

(A) Cross-correlation curves of αGalCer-AF488 and Dylight-633 labeled human LDL on day 1 and day 7, indicating stable complex formation after 7 days of storage at 4°C. (B) Cross-correlation curves of Dylight488-iGb3 and Dylight-633 labeled human LDL on day 1 and day 7, indicating stable complex formation after 7 days of storage at 4°C.

Supplemental fig 3. Lipid antigen concentration in different lipoprotein fractions

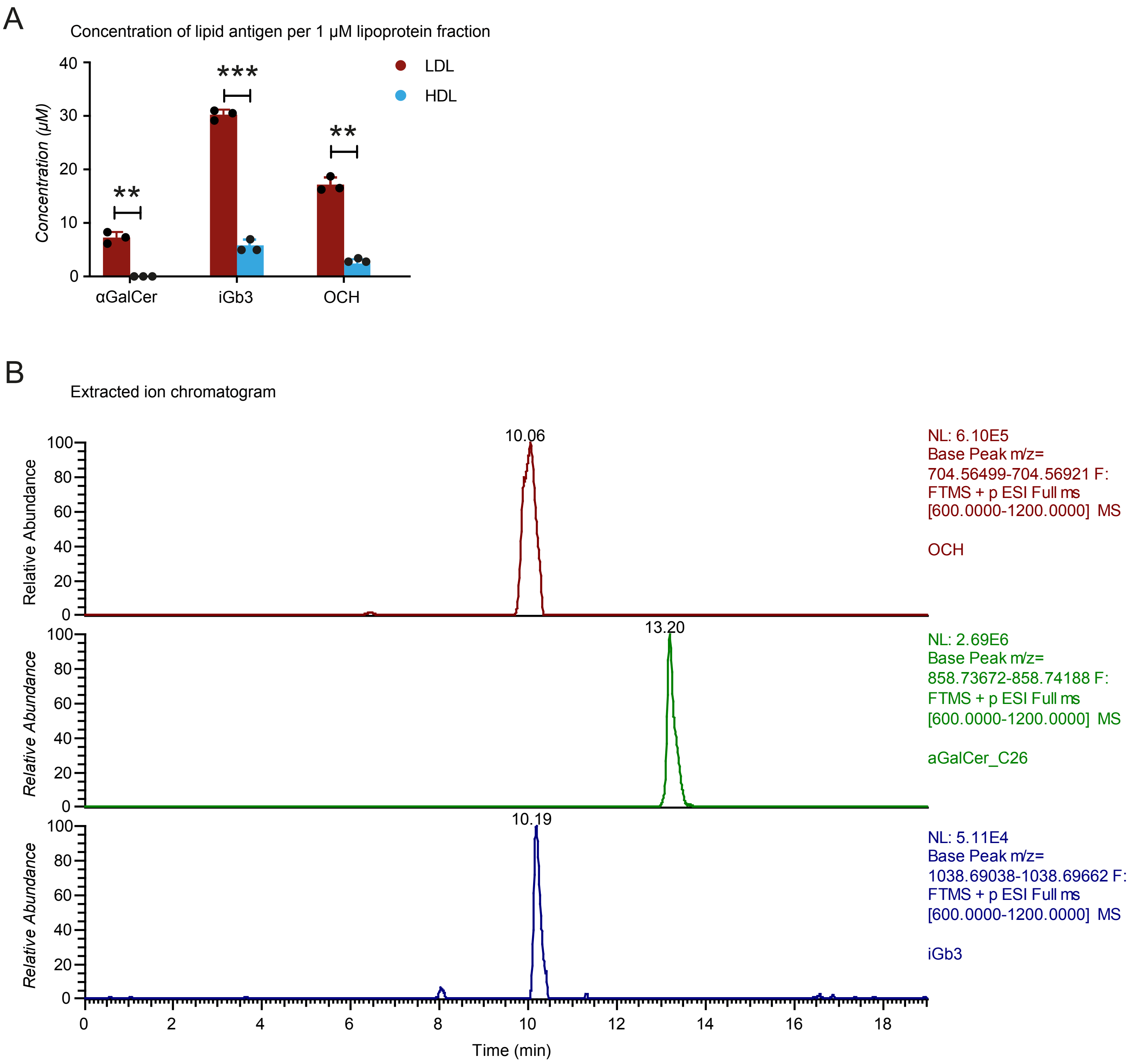

Supplemental figure 3.

(A) Concentration of lipid antigens in lipoprotein fractions LDL and HDL adjusted to 1 μM of lipoprotein fraction. (B) Extracted ion chromatogram of OCH, αGalCer and iGb3. Statistics: error bars represent mean ± SD. Independent t-test with Bonferroni correction for multiple comparisons (panel A). ** $P < 0.01$; *** $P < 0.001$.

Supplemental fig 4. iNKT cell activation by lipoprotein-αGalCer complexes in vivo

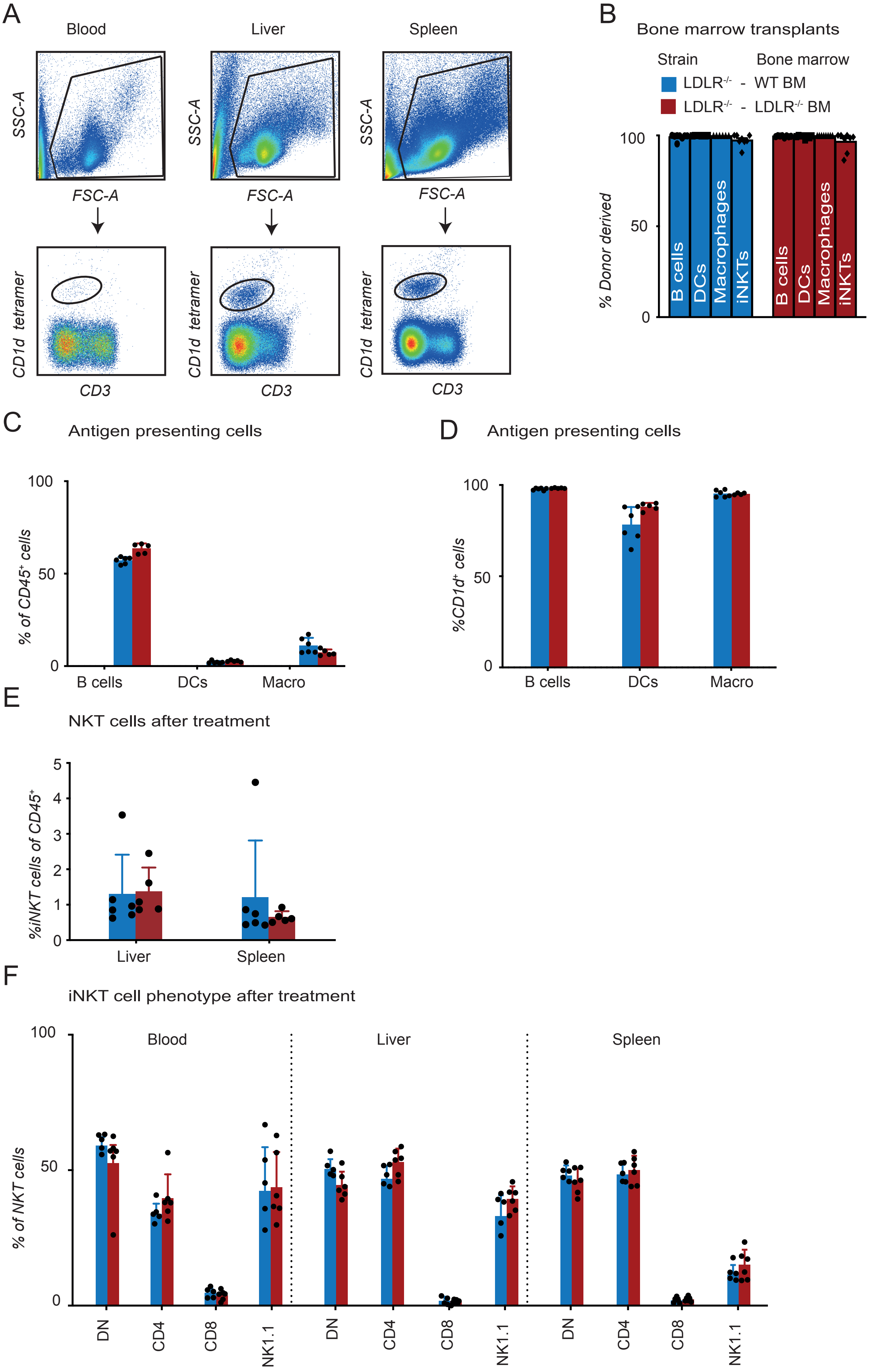

Supplemental figure 4. Lipoprotein-αGalCer complexes activate iNKT cells in vivo in a LDLR-mediated fashion.
(A) Representative flow cytometry and gating of blood, liver and spleen iNKT cells showing CD3 versus iNKT cell tetramer staining, upon selection of live single cells. FSC, forward scatter; SSC, side scatter. (B) Percentage of donor derived B cells, dendritic cells (DCs), macrophages and iNKT cells. (n = 11-12 per bone marrow chimera). (C) Antigen presenting cells in the bone marrow chimeras, as a percentage of live CD45+ cells. (n = 5-6). (D) CD1d expression on antigen presenting cells in the bone marrow chimeras. (n = 5-6). (E) Percentage of iNKT cells of live CD45+ cells extracted from liver and spleen 2 weeks post-treatment. (n = 5-6). (F) Percentage of CD4-CD8- (DN), CD4+, CD8+ and NK1.1+ iNKT cells in blood, liver and spleen 2 weeks post-treatment. (n = 5-6). Statistics: error bars represent mean ± SD. Mann-Whitney U tests (panel E), unpaired t tests (panel F).

Supplemental fig 5. Human LDLR mutations are associated with impaired iNKT cell activation

A

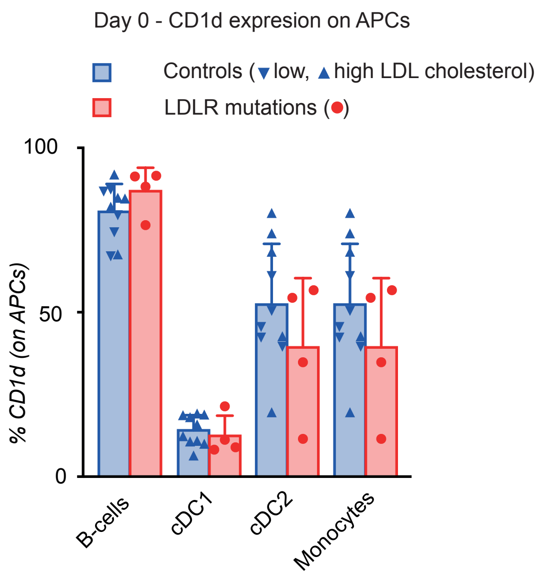

Supplemental figure 5.
(A) Flow cytometric analysis of CD1d expression on antigen presenting cells in LDLR-mutated PBMCs and controls.
(n = 10 controls and 4 patients). Blue downward triangles represent controls with low circulating LDL-cholesterol levels (≤ 2.5mmol/l, n = 5), while blue upward triangles represent controls with high circulating LDL-cholesterol levels (> 2.5mmol/l, n = 5), and red circles represent LDLR-mutated patients (n = 4).
Statistics: error bars represent mean ± SD. Independent t-test.
